# Building stronger primary care with AI: from innovation to implementation

**DOI:** 10.3399/BJGPO.2025.0285

**Published:** 2026-05-06

**Authors:** Heidi Smith, Lucy Smith, Yousef Yousef, Hajira Dambha-Miller

**Affiliations:** 1 Primary Care Research Centre, University of Southampton, Aldermoor Health Centre, Southampton, UK

**Keywords:** Primary care resilience, AI, Primary healthcare, General practitioners

## AI and non-clinical primary health staff: an overlooked opportunity

With the 2025 NHS 10-year plan calling for urgent system reform, the NHS must continue to identify and evaluate novel technologies that can relieve current service pressures and help mitigate the challenges posed by a globally ageing population with increasing multimorbidity.^1^ In primary care, the delivery of services depends not only on clinicians but also on a substantial non-clinical workforce, including practice managers and staff responsible for human resources, finance, information governance, and operational delivery. These roles are essential to the functioning of individual general practices and Primary Care Networks (PCNs), where they shape organisational processes, workforce deployment, and the implementation of new systems.

Despite their central role in organisational decision-making and service delivery, there is limited empirical research examining the use, perceptions, and governance of artificial intelligence (AI) among non-clinical primary care staff, from reception and care navigation to administrative coordination and practice management. This represents an important gap in the evidence base, as the successful adoption of AI is likely to be influenced by organisational, operational, and regulatory factors overseen by this workforce. This editorial therefore aims to highlight the relevance of non-clinical staff to AI implementation in primary care, and to outline why their perspectives are critical to understanding how AI technologies may be effectively and safely embedded within general practice.

Digital access tools such as the NHS App and commercial platforms (for example, MyGP by Huma) are increasingly positioned as the first point of contact between patients and general practice, aligning with the policy ambition for Modern General Practice.^2^ In practice, however, the operational workload generated by these systems is largely managed by non-clinical teams, particularly care navigation and triage staff, who spend substantial time reviewing online submissions, assessing urgency, and coordinating appointment capacity across practices and PCNs. AI-enabled triage tools, including conversational ‘digital front door’ systems, could automate aspects of this initial processing by directing patients to appropriate care pathways, thereby reducing administrative workload and supporting more timely access to care. One evaluation suggests that such tools could reduce administrative time by up to 43 minutes per staff member per day, although real-world impact will depend on local implementation and governance arrangements.^3^

Beyond access and triage, non-clinical staff play a central role in maintaining and acting upon clinical data held within primary care electronic medical records (EMRs), such as EMIS Web, TPP SystmOne, and Epic. Accurate and comprehensive clinical coding underpins continuity of care, service planning, audit, disease surveillance, research recruitment, and proactive interventions including screening and long-term condition reviews. It is also fundamental to the Quality and Outcomes Framework (QOF), which provides financial incentives to practices based on coded clinical activity.^4^ Incomplete or inconsistent coding can therefore lead to underreporting of activity and missed opportunities for practice income. Emerging AI tools, including natural language processing systems such as MedPromptExtract, can convert unstructured free text into structured coded data, potentially improving coding completeness and reliability.^5^ Ambient AI scribing technologies, such as Heidi Health, which is now widely used in the UK, may further support this process by generating contemporaneous documentation and coded outputs during consultations, reducing downstream workload for clinicians and administrative staff while improving the auditability of records.^6^

AI-driven data analytics may also support non-clinical staff in addressing inefficiencies in appointment utilisation. In 2019, unattended GP appointments (‘did not attends’ [DNAs]) were estimated to cost primary care £216 million annually, with approximately 7.2 million appointments missed each year.^7^ DNAs disproportionately affect older adults, individuals from socioeconomically deprived backgrounds, those with mental health conditions, and some minority ethnic groups, contributing to reduced access and widening health inequalities.^8^ Predictive AI tools, such as those developed by Deep Medical, use risk stratification to identify patients at higher risk of non-attendance and enable targeted interventions, including tailored messaging or dynamic scheduling.^9^ An NHS pilot reported a 30% reduction in DNAs over 6 months, allowing nearly 2000 additional appointments to be delivered.^10^ Such systems rely on non-clinical staff for oversight, configuration, and ethical deployment, underscoring their role in translating AI outputs into operational benefit.

AI platforms that integrate population health data, such as Abtrace, can support non-clinical teams by summarising outstanding actions across practice populations, including cancer screening, vaccinations, and long-term condition reviews, and by enabling automated recall and self-booking workflows.^11^ Remote monitoring and virtual care platforms, such as Huma, extend this approach by enabling continuous capture of physiological data and AI-assisted patient guidance between GP contacts.^2^ When effectively governed and integrated, such tools may enhance patient engagement and support proactive care; however, their implementation depends on non-clinical staff to manage data flows, information governance, and operational sustainability.

### Reimagining primary care with AI: from aspirational to operational

Figure 1 illustrates how AI in non-clinical primary care teams can shift services from reactive to proactive, improve data quality, cut costs, and maximise appointment availability. Piloting AI in operational roles offers a low-risk way for practices to gain benefits without disrupting clinical care. Evolving AI tools, such as Abtrace, could enhance coding, standardise data, improve auditability, and support proactive care while enabling better data flow across systems.^11^ In the future, federated learning could allow integrated records across primary, secondary, and community care without moving data, addressing governance and privacy concerns.

**Figure 1.**
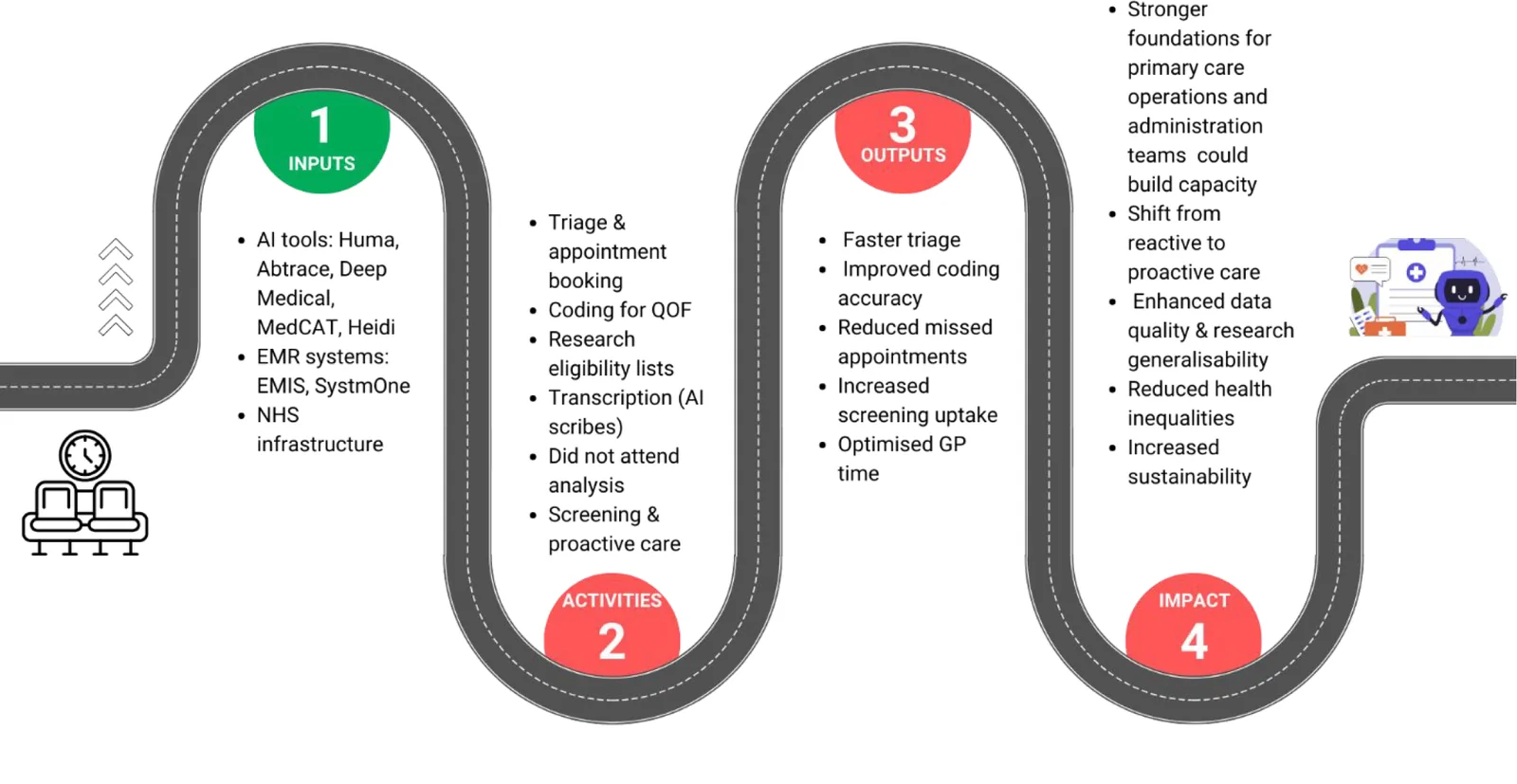
A roadmap for AI implementation into primary care non-clinical teams. QOF = Quality and Outcomes Framework.

Despite the potential operational benefits of AI in primary care, several barriers to implementation remain. Non-clinical staff often voice concerns that the growing use of AI in primary care may threaten their job security, with fears of being ‘replaced’ by automation. Yet emerging workforce analyses indicate that AI is more likely to reshape than remove these positions. Research highlights the development of new roles in AI oversight, implementation, and workflow redesign, suggesting that non-clinical staff will be central to the successful and safe integration of AI across primary care systems.^12^ Rather than diminishing non-clinical staff contribution, AI creates opportunities for role diversification, upskilling, and more meaningful involvement in guiding digital transformation within primary care.

The increasing commercialisation of AI adds further complexity, requiring that NHS partnerships with private developers balance innovation against considerations of data ownership, equity of access, and value for public investment. Robust procurement processes, transparent evaluation of cost-effectiveness, and clear governance frameworks will be essential to ensure that AI adoption prioritises patient benefit rather than primarily serving commercial interests. Emerging non-clinical roles in AI governance and data oversight could provide valuable support in maintaining these standards and strengthening organisational accountability.

From an operational perspective, current AI systems also face technical limitations, particularly in triage and risk stratification, where context-sensitive reasoning is required to identify complex or atypical presentations. Under an augmented intelligence model, non-clinical care navigation and operational teams will play a critical role in overseeing, validating, and acting upon AI outputs, supported by clear standard operating procedures to ensure safety, consistency, and accountability. Introducing AI initially within administrative and organisational functions offers a potentially lower risk approach to adoption, allowing general practice to strengthen its operational foundations while preserving core clinical relationships. Careful, staged implementation that recognises the central role of non-clinical staff will be key to translating AI in primary care from policy aspiration into sustainable practice, as depicted in Figure 2.

**Figure 2.**
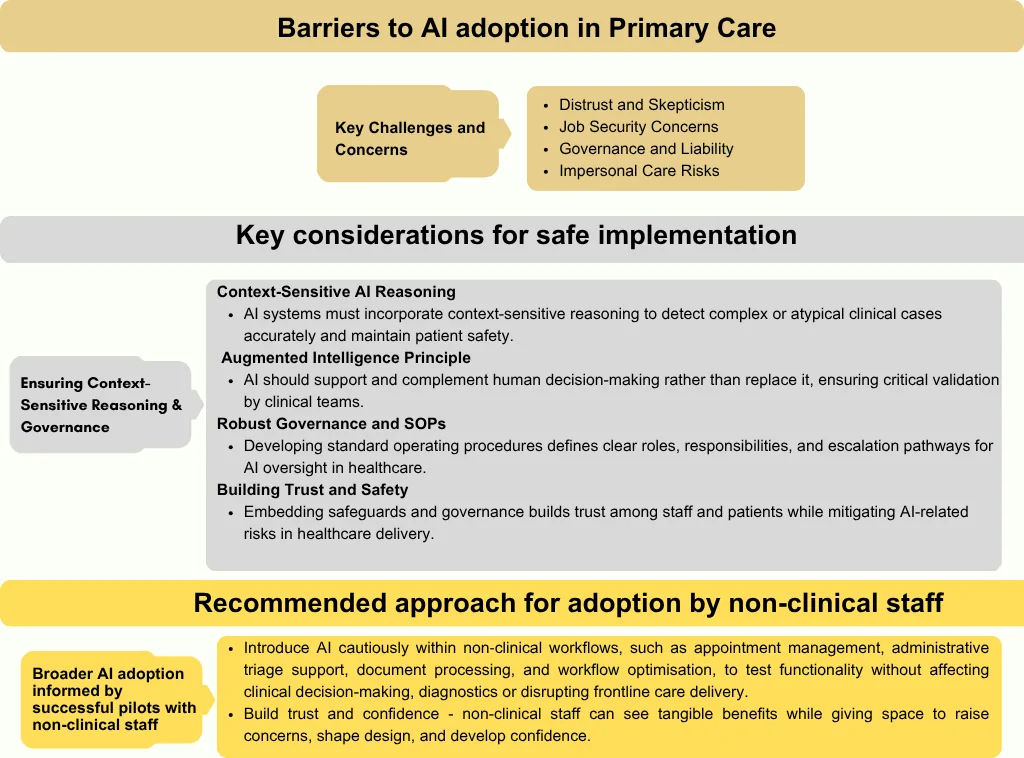
Barriers and pathways for AI adoption in primary care. SOPs = standard operating procedures.

## Data

All articles utilised are publicly available.
